# β1 integrin- and JNK-dependent tumor growth upon hypofractionated radiation

**DOI:** 10.18632/oncotarget.10522

**Published:** 2016-07-11

**Authors:** Aejaz Sayeed, Huimin Lu, Qin Liu, David Deming II, Alexander Duffy, Peter McCue, Adam P. Dicker, Roger J. Davis, Dmitry Gabrilovich, Ulrich Rodeck, Dario C. Altieri, Lucia R. Languino

**Affiliations:** ^1^ Prostate Cancer Discovery and Development Program, Philadelphia, PA, USA; ^2^ Department of Cancer Biology, Thomas Jefferson University, Philadelphia, PA, USA; ^3^ Molecular and Cellular Oncogenesis Program, The Wistar Institute, Philadelphia, PA, USA; ^4^ Department of Pathology, Thomas Jefferson University, Philadelphia, PA, USA; ^5^ Department of Radiation Oncology, Thomas Jefferson University, Philadelphia, PA, USA; ^6^ Program in Molecular Medicine, University of Massachusetts Medical School, Worcester, MA, USA; ^7^ Howard Hughes Medical Institute, University of Massachusetts Medical School, Worcester, MA, USA; ^8^ Translational Tumor Immunology Program, The Wistar Institute, Philadelphia, PA, USA; ^9^ Department of Dermatology and Cutaneous Biology, Thomas Jefferson University, Philadelphia, PA, USA; ^10^ Tumor Microenvironment and Metastasis Program, The Wistar Institute, Philadelphia, PA, USA

**Keywords:** TRAMP mice, prostate cancer, β1 integrins, FAK, insulin-like growth factor receptor

## Abstract

Radiation therapy is an effective cancer treatment modality although tumors invariably become resistant. Using the transgenic adenocarcinoma of mouse prostate (TRAMP) model system, we report that a hypofractionated radiation schedule (10 Gy/day for 5 consecutive days) effectively blocks prostate tumor growth in wild type (β1^wt^ /TRAMP) mice as well as in mice carrying a conditional ablation of β1 integrins in the prostatic epithelium (β1^pc-/-^ /TRAMP). Since JNK is known to be suppressed by β1 integrins and mediates radiation-induced apoptosis, we tested the effect of SP600125, an inhibitor of c-Jun amino-terminal kinase (JNK) in the TRAMP model system. Our results show that SP600125 negates the effect of radiation on tumor growth in β1^pc-/-^ /TRAMP mice and leads to invasive adenocarcinoma. These effects are associated with increased focal adhesion kinase (FAK) expression and phosphorylation in prostate tumors in β1^pc-/-^ /TRAMP mice. In marked contrast, radiation-induced tumor growth suppression, FAK expression and phosphorylation are not altered by SP600125 treatment of β1^wt^ /TRAMP mice. Furthermore, we have reported earlier that abrogation of insulin-like growth factor receptor (IGF-IR) in prostate cancer cells enhances the sensitivity to radiation. Here we further explore the β1/IGF-IR crosstalk and report that β1 integrins promote cell proliferation partly by enhancing the expression of IGF-IR. In conclusion, we demonstrate that β1 integrin-mediated inhibition of JNK signaling modulates tumor growth rate upon hypofractionated radiation.

## INTRODUCTION

Integrin-mediated adhesion of cancer cells to the extracellular matrix regulates the cellular response to ionizing radiation [1-3]. Our laboratory and others have shown that integrins regulate the response to radiation by modulating the activity of c-Jun NH2-terminal kinase (JNK) [3-5], a member of the MAPK family, also known as stress-activated protein kinase [6]. Integrin regulation of JNK signaling is complex; JNK has been shown to be either activated by β1 or αv integrins in head and neck [4] and in nasopharyngeal cancer [5] or suppressed by β1 integrins in prostate cancer as reported in our *in vivo* study [3].

While recent advances in radiotherapy have enabled precise targeting of tumor tissue, recurrence after radiotherapy, however, remains a concern. Many factors may lead to the failure of radiotherapy and to recurrence (Reviewed in [7]) including enhanced DNA repair, activation of tumor cell survival pathways, and inhibition of programmed cell death as well as the presence of a subpopulation of cancer stem cells that are inherently resistant to radiation (Reviewed in [8]). The conventional external beam radiation therapy used in the clinic ranges from 75.6 to 81.0 Gy of radiation divided into 1.8- to 2.0 Gy fractions, and is carried out daily between 7 and 9 weeks (Reviewed in [9]). Recently, moderate (<35 fractions) and extreme (<5 fractions) hypofractionated radiation therapy has been reported to yield more favorable results than conventional regimens (2 Gy/fraction), both in terms of biochemical response and toxicity [10]. However, there is no consensus in the scientific community whether hypofractionated radiation significantly reduces biochemical and/or clinical disease failure [11]. Thus, in the current study, we have investigated the effect of hypofractionated high dose radiation administered at shorter intervals (mice are irradiated with a total dose of 50 Gy, carried out in fractionated doses of 10 Gy, consecutively for 5 days). This simulates the approach proposed for clinical use in an effort to alleviate patient inconvenience and reduce health care costs (Reviewed in [9]). An improved understanding of the mechanisms involved in radiation-induced tumor regression may ultimately provide novel strategies of intervention in the treatment of human malignancies.

Using this hypofractionated radiation approach, we have tested the effect of a JNK inhibitor SP600125 (SP) on radiation response in wild type (β1^wt^ /TRAMP) mice as well as in mice carrying a conditional ablation of β1 integrins in the prostatic epithelium (β1^pc-/-^ /TRAMP). SP is a reversible ATP-competitive inhibitor of JNK that blocks all three JNK isoforms with similar potency [12]. Its specificity is attributed to the fact that it effectively occupies the hydrophobic pocket of the ATP binding site in JNK1 and variations of crucial hydrophobic residues in other MAP kinases make JNK a selective target [13]. SP has been reported to be a selective JNK inhibitor [14] and its effectiveness against JNK *in vivo* has been widely reported [15-18].

In the present study, we demonstrate that the effects of JNK inhibition are contingent upon β1 integrin expression. We studied the signaling interface between β1 integrins and the type-1 insulin-like growth factor receptor (IGF-IR), a trans-membrane tyrosine-kinase receptor, known to play an essential role in the development and progression of cancer by regulating cell proliferation, differentiation, apoptosis and metastasis [19]. Like β1 integrins, IGF-IR signaling has been reported to mediate resistance to radiotherapy [20, 21]. Together, these receptors play a concerted role in radio-resistance of cancer cells [3] and unraveling the nature of these interactions is expected to contribute not only to understanding the mechanisms of resistance, but also in the identification of novel therapeutic targets.

Here, we have studied β1-mediated downstream signaling *in vivo* in prostate cancer resistance to hypofractionated radiation. We report that JNK inhibition compromises the beneficial effects of radiation therapy in TRAMP mice carrying conditional ablation of β1 (β1^pc-/-^ /TRAMP), and results in a significant increase in prostate tumor growth associated with increased FAK expression and activity in these tumors.

## RESULTS

### JNK inhibition in β1^pc-/-^ TRAMP mice irradiated in the lower pelvis leads to prostate cancer progression

**Figure 1:**
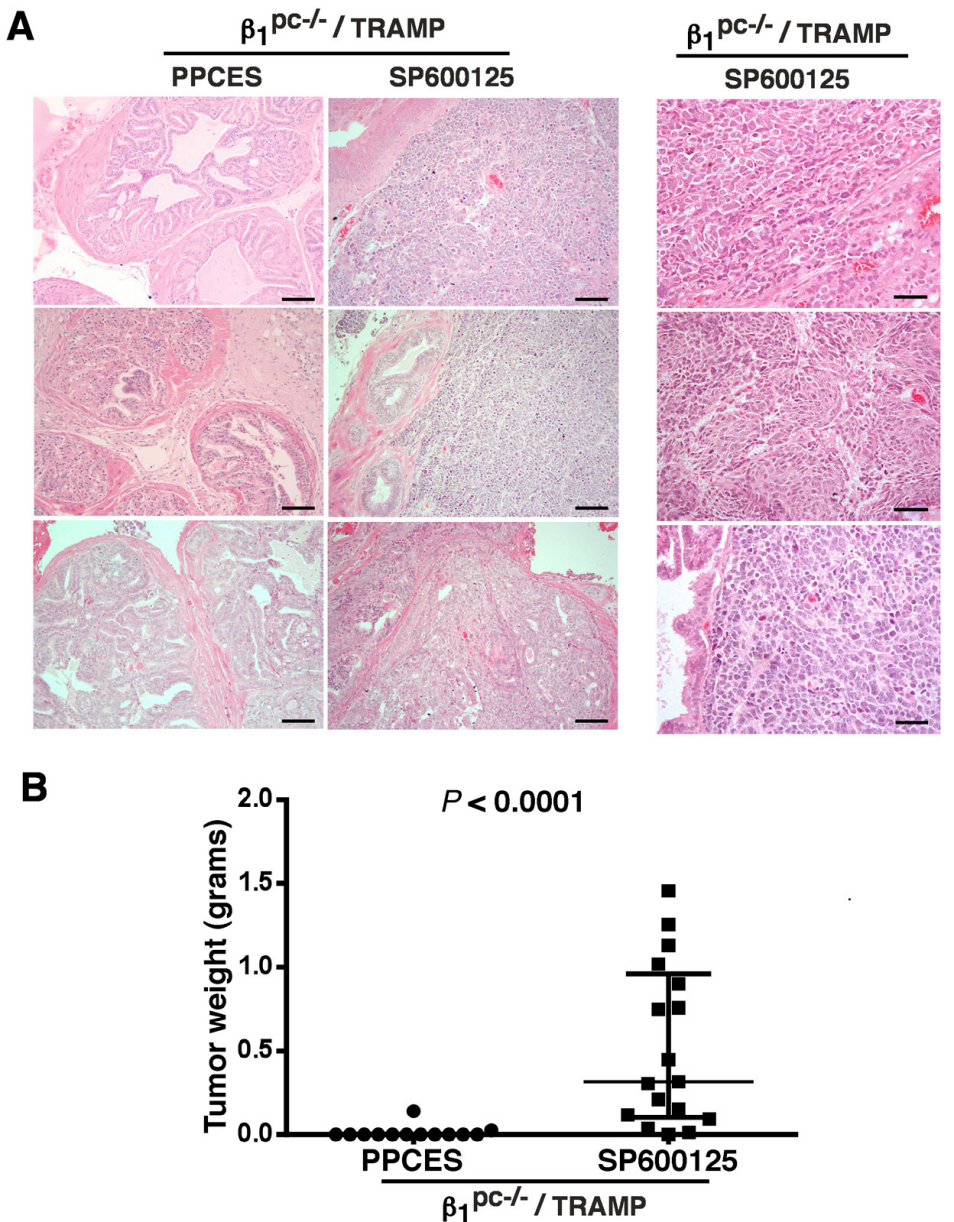
JNK inhibition counteracts the effect of irradiation and supports aggressive prostate growth in β1^-/-^ TRAMP mice **A.** Histopathological analysis after Hematoxylin and Eosin (H&E) staining of prostate tissue from irradiated β1^pc-/-^ /TRAMP mice. At the age of 20 weeks, mice were subjected to IP injections of vehicle PPCES or inhibitor SP600125, administered 3 times/week for 10 weeks. Two hours after the first IP injection with either PPCES or SP600125, the lower pelvises of the mice were irradiated with a hypofractionated radiation regimen that consisted of 10 Gy fractions (total dose 50 Gy) administered for 5 consecutive days. Mice were euthanized at 35 weeks of age and prostate tissues were analyzed for tumor progression. Representative H&E images of tissues from control or treated mice are shown (left panels). Scale bar 100 μm. The right panel shows representative H&E high magnification images of prostate tumors from irradiated and SP600125-treated β1^pc-/-^ /TRAMP mice depicting aggressive histopathology. Scale bar 50 μm. **B.** The tumor mass distribution in irradiated β1^pc-/-^ TRAMP mice injected with PPCES vehicle or SP600125-treated is shown. Median tumor weight with interquartile range and individual data points are plotted. PPCES group *n* = 13 mice, SP600125 group *n* = 17 mice. A statistically significant increase in tumor mass is found in the SP600125-treated cohort as compared to the cohort injected with PPCES. *P* < 0.0001 (Wilcoxon-Mann-Whitney two-sided test).

We have previously demonstrated that conditional ablation of β1 integrins significantly improves survival and delays prostate cancer progression in response to lower pelvis irradiation in TRAMP mice [3]. To assess the importance of JNK activation in response to radiation, 20 week-old β1^pc-/-^ /TRAMP mice were treated with hypofractionated radiation regimen that consisted of 10 Gy fractions for 5 consecutive days (total 50 Gy). In conjunction with radiation treatment, intraperitoneal (IP) injections of SP and PPCES (PP) were administered three times/week and were continued for 9 additional weeks followed by euthanasia at 35 weeks of age. This irradiation strategy led to effective tumor suppression in β1^pc-/-^ /TRAMP mice. JNK inhibition in these mice markedly compromises the effect of radiation and results in increased tumor load at the end of the treatment period. Histopathological analysis of prostate tissues from irradiated β1^pc-/-^ /TRAMP mice treated with SP consistently reveals aggressive pathological characteristics when compared to controls injected with PP which show *in situ* carcinoma characterized by marked papillary and cribriform epithelial proliferation with acinar expansion (Figure 1A, left panels). High magnification images of prostate tissues from SP-treated mice (Figure 1A, right panel) specifically reveal invasive carcinoma composed of anaplastic epithelioid cells growing in sheets and nests. Individual cells show marked cytologic dysplasia and areas of tissue necrosis within the expanded acini. Analysis of total tumor mass in irradiated mice injected with either PP (*n* = 13) or SP (*n* = 17) indicates that SP treatment significantly increases tumor mass in β1^pc-/-^ /TRAMP mice, *P* < 0.0001 (Figure 1B). These results suggest that the therapeutic effect of β1 abrogation in response to irradiation is offset by JNK inhibition.

### JNK inhibition in β1^wt^ /TRAMP mice does not interfere with radiation-induced tumor suppression

**Figure 2:**
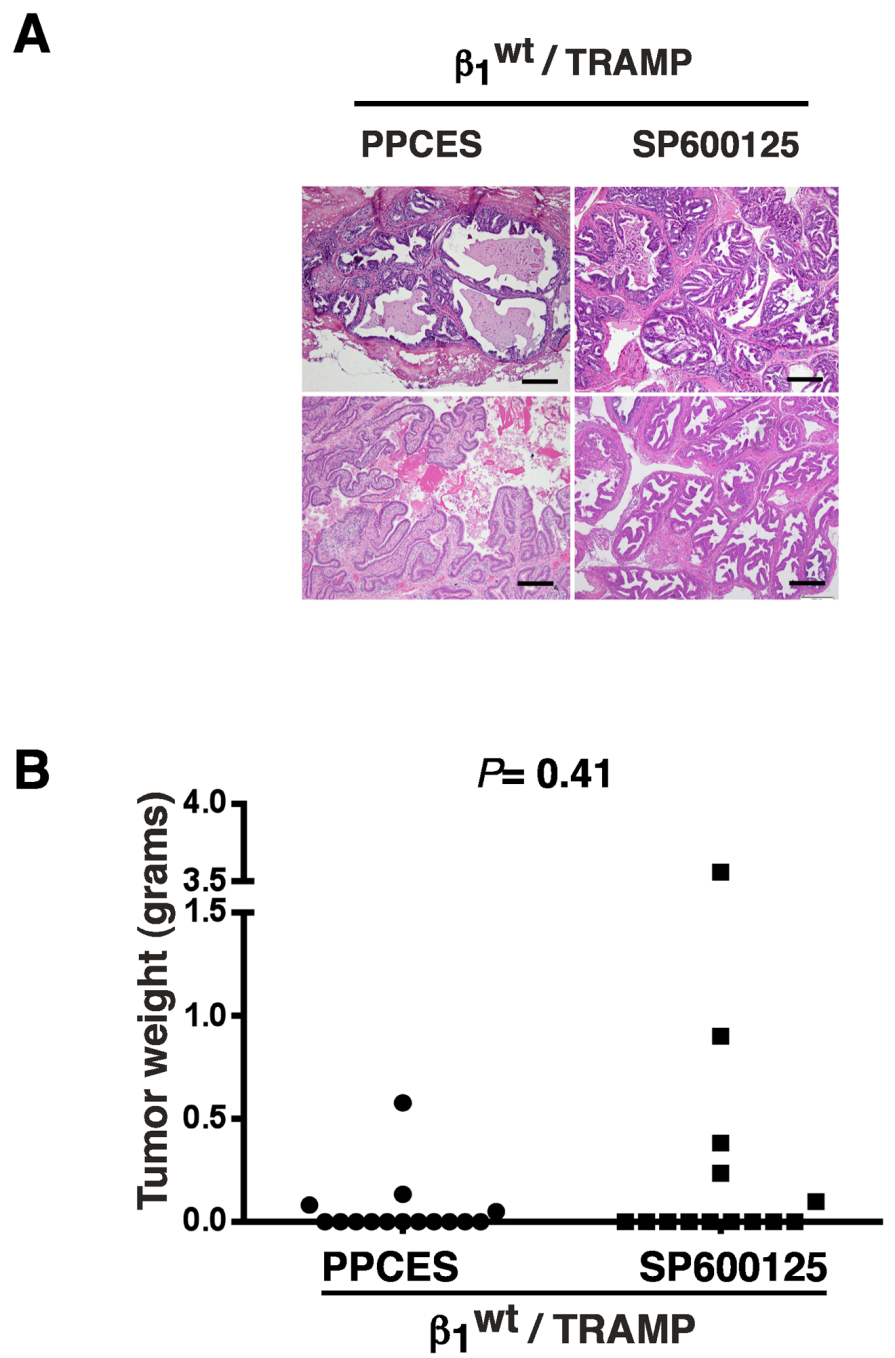
JNK inhibition in β1 /TRAMP mice does not offset the effect of radiation on tumor growth **A.** Histopathological analysis of irradiated tumors from β1^wt^ /TRAMP mice treated as described in Figure 1. Representative H&E images of tissues from control or treated mice are shown. Scale bar 100 μm. **B.** The tumor mass distribution in lower pelvis-irradiated β1^wt^ /TRAMP mice injected with PPCES or SP600125 is shown. Median tumor weight and individual data points are plotted. PPCES group *n* = 15 mice, SP600125 group *n* = 14 mice. No statistically significant differences in tumor mass are found between SP and vehicle treated β1^wt^ /TRAMP cohorts. *P* = 0.4094 (Wilcoxon-Mann-Whitney two-sided test).

Since we have previously demonstrated that β1 integrins suppress radiation-induced JNK activation [3], the JNK inhibitor was not expected to elicit any changes upon prostate irradiation in mice expressing wild type β1 (β1^wt^ /TRAMP). To test whether JNK inhibition influences radiation resistance in β1^wt^ /TRAMP prostate, we carried out irradiation and PP or SP injections in β1^wt^ /TRAMP mice as described above in Figure 1. Histopathological analysis of prostate tissues from irradiated β1^wt^ /TRAMP mice injected with either PP or SP does not show any tumor growth (Figure 2). Prostate tissues from SP-treated β1^wt^ mice show marked papillary and cribriform epithelial hyperplasia with high-grade dysplasia (Figure 2A, right panel). In some glands, the changes are equivalent to *in situ* carcinoma; however, invasive carcinoma is not observed in this group. The prostatic tissues from the PP group show a spectrum of phenotypes including papillary hyperplasia with mild atypia, papillary and cribriform hyperplasia with high-grade dysplasia and combined epithelial and stromal hyperplasia with minimal cytologic atypia (Figure 2A, left panel). The differences in tumor mass between PP (*n* = 15) and SP (*n* = 14) cohorts are not significant, *P* = 0.41 (Figure 2B). These data imply that JNK inhibition does not modify the radiation response in β1^wt^ tumors.

**Figure 3:**
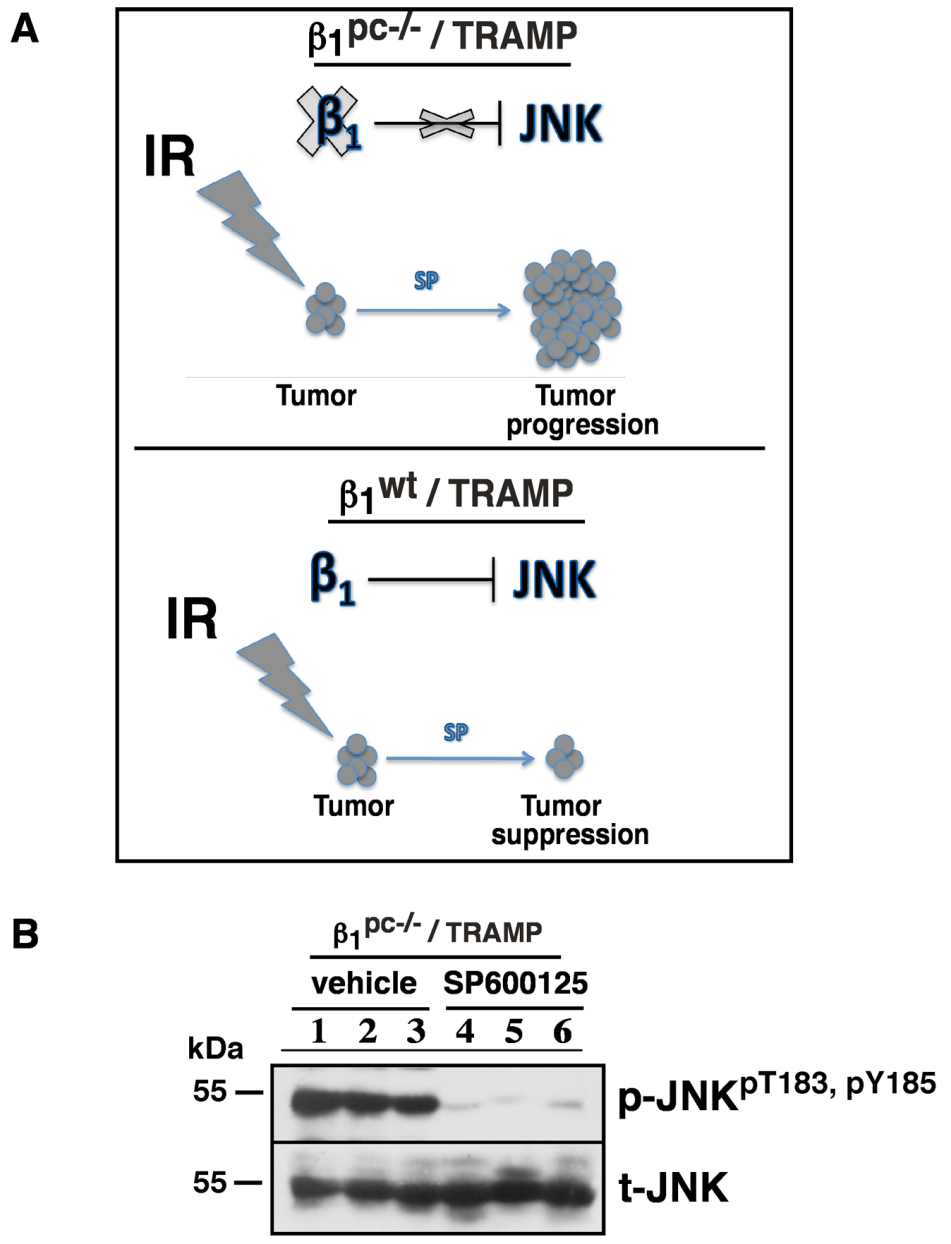
JNK signaling is crucial for radiation-dependent tumor suppression in the absence of β1 integrins **A.** Schematic representation showing that the effect of radiation is offset by JNK inhibition in β1^pc-/-^/TRAMP mice (upper panel). In contrast, the effect of radiation persists in SP600125-treated β1^wt^ /TRAMP mice (lower panel). **B.** Immunoblot analysis shows the total and JNK^pT183, pY185^ levels in irradiated prostate tissues of β1^pc-/-^ / TRAMP mice treated as in Figure 1.

Collectively, these data show that JNK inhibition counteracts the effect of radiation therapy in the absence of β1 integrins and accelerates tumor growth and progression as indicated in our schematic drawing in Figure 3A. This is consistent with our earlier findings that JNK activation is suppressed by β1 integrins in prostate cancer cells [3] and with our new observation that exogenous JNK inhibition does not affect tumor growth in β1^wt^ mice. To confirm SP-mediated JNK suppression *in vivo*, prostate tissues isolated from irradiated controls (PP cohort) and irradiated plus SP-treated β1^pc-/-^ /TRAMP cohort were evaluated for JNK function. Our results demonstrate that SP significantly blocks the phosphorylation of JNK in prostate tissues (Figure 3B).

### JNK inhibition *in vivo* up-regulates the expression and activation of FAK in irradiated β1^pc-/-^ prostate tumors

**Figure 4:**
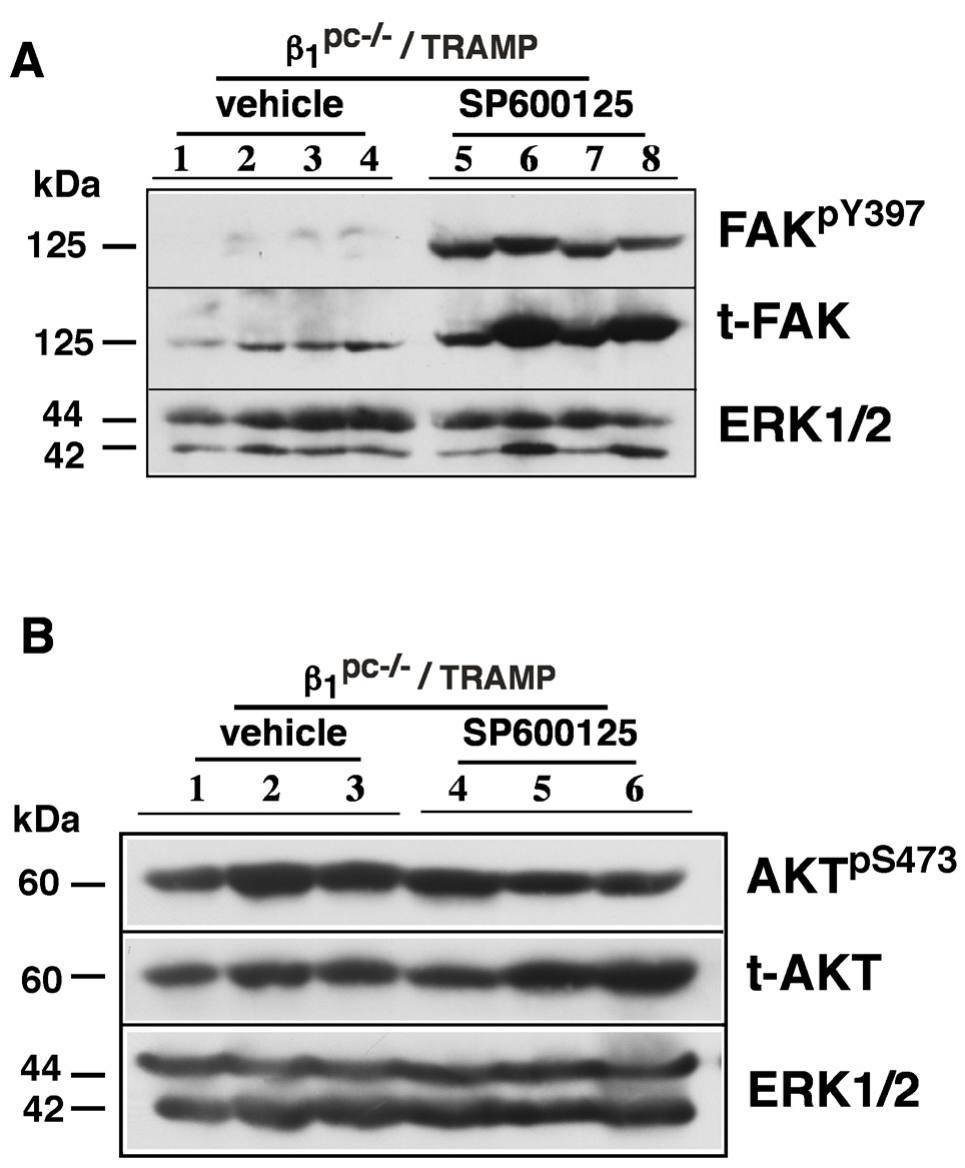
Tumor growth upon irradiation and JNK inhibition in β1^-/-^/TRAMP mice is associated with enhanced FAK signaling **A.** Induction of FAK expression and phosphorylation in prostate tumors from β1^pc-/-^ /TRAMP mice. Immunoblot analysis of prostate tissues from β1^pc-/-^ /TRAMP mice treated as described in Figure 1. Prostate tissues were snap frozen. Tissues were homogenized and lysates analyzed by SDS-PAGE using Abs against FAK^pY397^ and total FAK. ERK1/2 was used as a loading control. **B.** Immunoblotting analysis of prostate tissues from β1^pc-/-^ /TRAMP mice showing AKT^pS473^ and total AKT in prostate tissues as described above in Figure 4A. ERK1/2 was used as a loading control.

**Figure 5:**
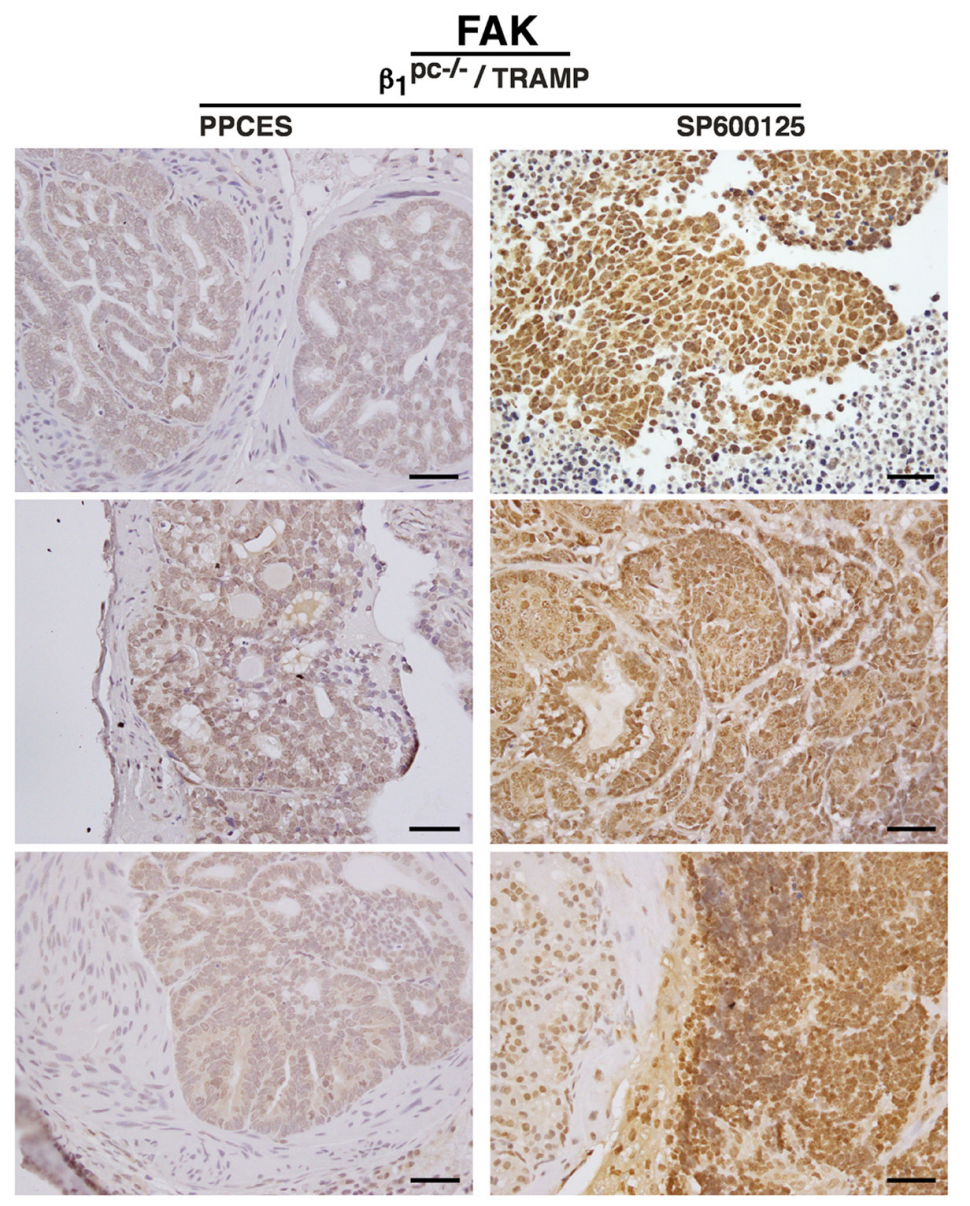
Irradiation coupled with JNK inhibition in β1^-/-^ /TRAMP mice leads to increased levels of nuclear FAK in tumor cells FAK expression profile in paraffin-embedded tissue sections from irradiated and SP600125-treated β1^pc-/-^ /TRAMP mice are shown. Ten specimens in control PPCES and SP600125-treated cohorts were analyzed. Representative expression profiles are shown here. Scale bar 50 μm.

FAK is known to support tumor growth and metastasis [22], and in TRAMP mice reportedly contributes to the development of neuroendocrine carcinoma [23]. Consistent with its role in aggressive forms of the disease, FAK signaling has been associated with radiation resistance [24]. To investigate if β1 integrins modulate FAK signaling in the presence of radiation, we evaluated FAK expression and activity profiles in our model system. Prostate tissues from irradiated and SP- treated β1^pc-/-^ /TRAMP mice were analyzed for the expression and phosphorylation of FAK. Our results demonstrate that JNK inhibition by SP selectively leads to the up-regulation of FAK expression and phosphorylation (Figure 4A). No changes in AKT phosphorylation, however, are observed in these tissues (Figure 4B). Consistent with FAK phosphorylation, we also observe induction of tyrosine kinase Src expression and phosphorylation upon JNK inhibition in tissues from β1^pc-/-^ /TRAMP mice (data not shown). In order to study the histological localization of FAK, prostate tissues from SP-treated β1^pc-/-^ /TRAMP mice were analyzed for FAK expression patterns by immunohistochemistry. Ten specimens in the PP and SP cohorts were studied for FAK intensity and scored. Our data indicate that SP-treated specimens show relatively higher FAK intensity and enhanced nuclear localization in comparison to the PP-control cohort (Table 1 and Figure 5). These data highlight the role of β1 and JNK signaling in FAK regulation and strongly suggest a role for FAK in β1-dependent radiation resistance of prostate cancer.

**Table 1:** Expression profile and localization of FAK in prostate tissues from irradiated and/or SP-treated β1^pc-/-^ / TRAMP mice

Mouse Number	JNK inhibitor	Histopathology	FAK intensity		FAK Positive Fields [Percentage]
			Nuclear	Cytoplasmic	
1	+	Invasive carcinoma	3	-	100%
2	+	Aggressive high grade tumor, neuroendocrine phenotype	3	-	100%
3	+	High grade tumor, carcinoma in situ	3	-	100%
4	+	High grade tumor	3	-	100%
5	+	Carcinoma in situ and high grade PIN	3	-	100%
6	+	High grade tumor	3	1	100%
7	+	High grade tumor	3	3	100%
8	+	Aggressive high grade tumor, high grade PIN, invasive carcinoma, unusual papillary cells	2-3	2-3	90%
9	+	High grade aggressive tumor	3	3	80%
10	+	High grade tumor	2-3	2-3	80%
11	_	Carcinoma in situ, benign, PIN	1-2	1-2	100%
12	_	Well contained high grade PIN	1-2	1-2	90%
13	_	Carcinoma in situ, high grade PIN	1-2	1-2	80%
14	_	Benign, atypical to well defined PIN	1	1	70%
15	_	PIN, tumor plus dysplasia	1	1	60%
16	_	No tumor, abscess	1	-	50%
17	_	Carcinoma in situ, locally invasive, high grade tumor	2	1	40%
18	_	Focal high grade PIN, carcinoma in situ, papillary hyperplasia	1-2	1-2	40%
19	_	Normal glandular morphology	1	1	30%
20	_	Typical hyperplasia, no tumor	1	1	20%

At the age of 20 weeks, β1^pc-/-^/TRAMP mice were subjected to IP injections of vehicle PP (-) or inhibitor SP (+), administered 3 times/week for 10 weeks. Two hours after the first IP injection with either PP or SP, the lower pelvises of the mice were irradiated with a hypofractionated radiation regimen that consisted of 10 Gy fractions (total dose 50 Gy) administered for 5 consecutive days. Mice were euthanized at 35 weeks of age and paraffin-embedded formalin-fixed prostate tissues were analyzed for FAK expression patterns. Histological features of 10 PP and 10 SP tissues are shown. “Percentage positive areas” at x40 optical magnification represents the number of cytoplasmic and/or nuclear FAK-positive regions over ten independent areas in each specimen analyzed. An optical region of a sample that showed more than 50% of cells with positive FAK staining was scored as FAK positive; FAK expression was scored using a 1-3 arbitrary scale in the FAK intensity column. -, indicates undetectable levels.

### JNK inhibition *in vivo* fails to enhance FAK/AKT signaling upon irradiation in β1^wt^ /TRAMP mice

**Figure 6:**
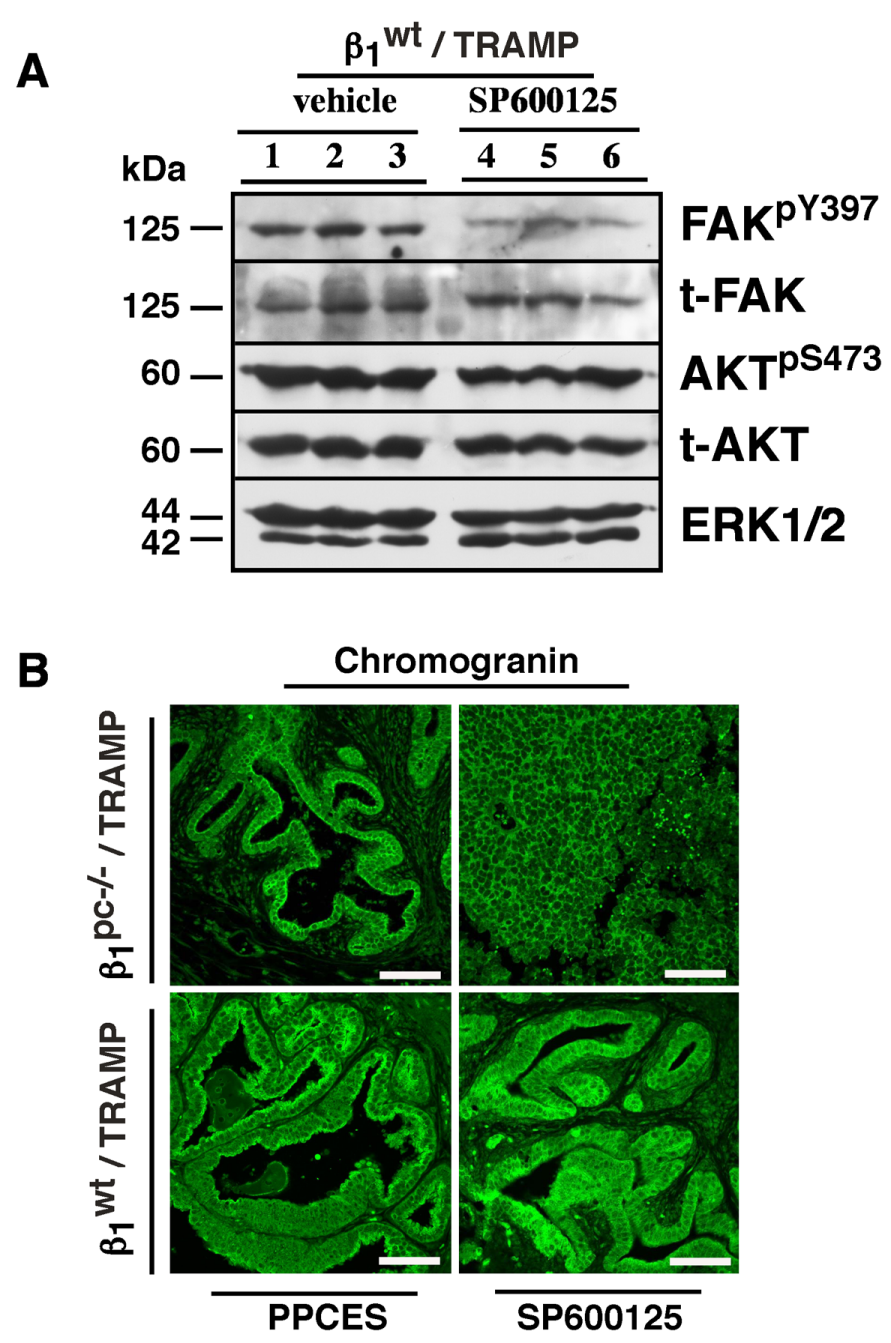
FAK/AKT signaling is not altered upon lower pelvis irradiation and JNK inhibition in β1/TRAMP mice **A.** Immunoblot analysis of prostate tissues from β1^wt^ /TRAMP mice treated as described in Figure 1. Frozen tissues were homogenized and lysates analyzed by SDS-PAGE using antibodies against total FAK, FAK^pY397^, total AKT and AKT^pS473^. ERK1/2 was used as a loading control. **B.** Neuroendocrine differentiation in response to irradiation and JNK inhibition in either β1^pc-/-^ /TRAMP or β1^wt^ /TRAMP prostate tissues. Number of mice analyzed: PPCES group β1^pc-/-^ /TRAMP, *n* = 5; SP600125 group β1^pc-/-^ /TRAMP, *n* = 3; PPCES group β1^wt^ /TRAMP, *n* = 3 and SP600125 group β1^wt^ /TRAMP, *n* = 3. Representative images reflecting the expression profile of chromogranin in β1^pc-/-^ or β1^wt^ TRAMP mice. Prostate tissues from β1^pc-/-^ or β1^wt^ /TRAMP mice treated as in Figure 1A above were fixed; sections were stained with a chromogranin Ab and processed for immunofluorescence. Counterstaining was done with DAPI not shown and expression profiles were studied by confocal microscopy. Scale bar 100 μm.

Since JNK inhibition leads to the induction of FAK signaling in β1^pc-/-^ /TRAMP prostate tumors, we assessed the FAK profile in β1^wt^ /TRAMP prostate tissues. β1 integrins are known to regulate FAK signaling [4] and we have previously demonstrated that β1 integrins suppress radiation-induced JNK signaling in prostate cancer [3]. Consistent with a dominant role of β1 integrins in suppressing JNK1 activation in β1^wt^ /TRAMP mice, JNK inhibition in β1^wt^ /TRAMP prostate tissues does not enhance FAK signaling (Figure 6A); we also observe that JNK inhibition in β1^wt^ /TRAMP prostate tissues does not enhance AKT signaling. Conversely, our results indicate that JNK-inhibition dependent FAK up-regulation in prostate tumors selectively occurs in the absence of β1 integrins. We also investigated the expression of a neuroendocrine marker, chromogranin, in β1^pc-/-^ and β1^wt^ /TRAMP prostate tissues. Chromogranin expression is detected in both β1^pc-/-^ and β1^wt^ tissues regardless of JNK inhibition implying that β1, JNK or FAK signaling does not influence chromogranin expression in our model (Figure 6B).

### β1 integrins regulate IGF-IR expression to enhance prostate cancer cell proliferation

**Figure 7:**
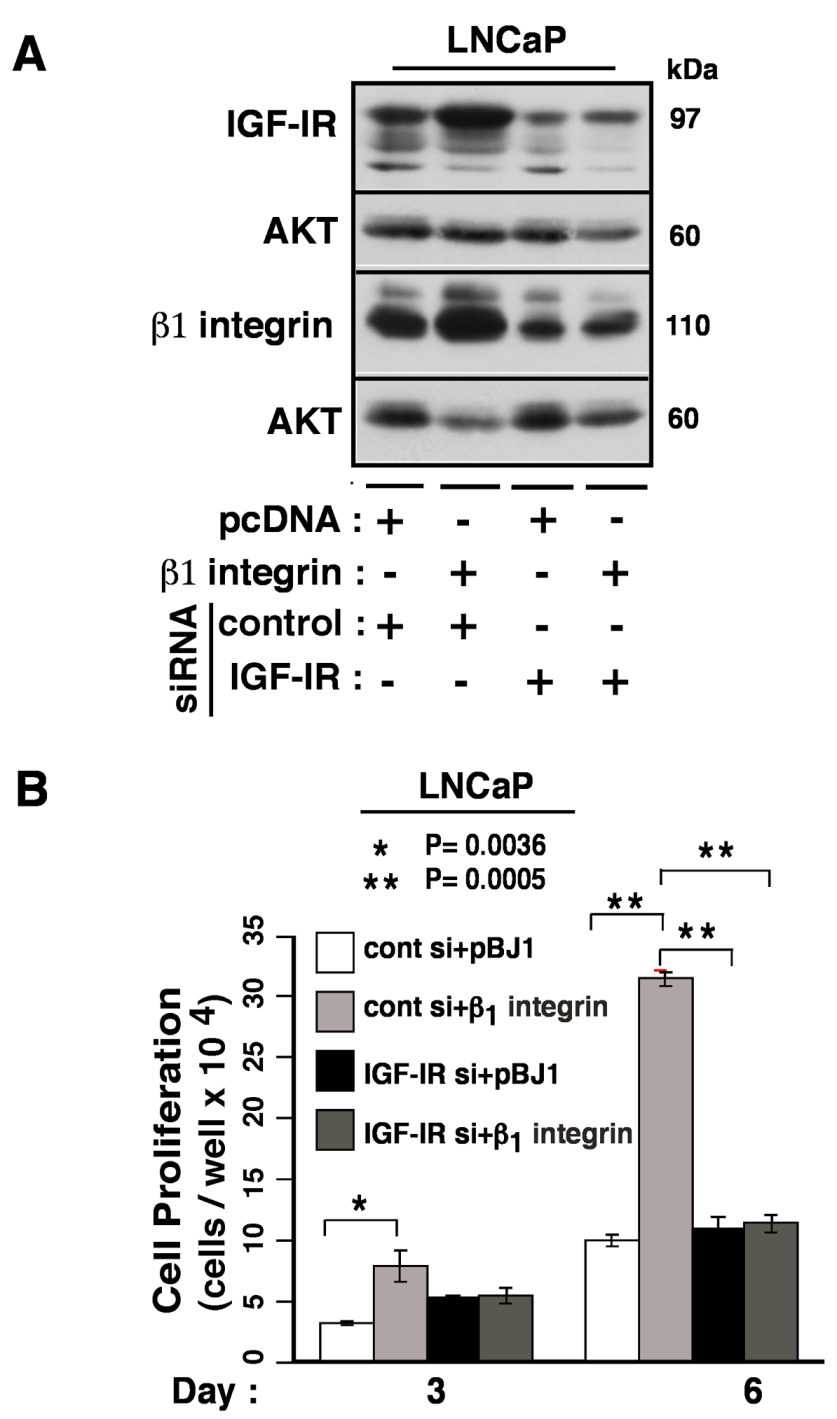
β1 integrins promote cancer cell proliferation by regulating the expression of IGF-IR **A.** Exogenous β1 induction leads to the up-regulation of IGF-IR expression. LNCaP cells were transiently transfected with either pBJ1 or β1-pBJ1 constructs, together with control or IGF-IR siRNA; 48 hours later, cell lysates were analyzed by immunoblotting analysis for expression of the β1 integrin subunit and IGF-IR. AKT was used as a loading control. **B.** Exogenous β1 induction enhances cancer cell proliferation, which is blocked by IGF-IR knockdown. LNCaP cells transfected as in Figure 7A were replated in equal numbers in culture dishes and allowed to grow for either 3 or 6 days followed by live cell counting. Each assay was performed in triplicate and asterisk signs represent statistical significance.

We have previously reported that β1 integrin expression is regulated by IGF-IR, a receptor known to promote resistance to radiation, and that abrogation of IGF-IR in prostate cancer cells leads to the loss of β1 integrins by proteasomal degradation [25]. Here we investigate whether β1 integrins affect IGF-IR expression. LNCaP cells were transiently transfected with β1-pcDNA together with control or IGF-IR siRNA to evaluate the expression of IGF-IR. Our results demonstrate that over-expression of β1 leads to the up-regulation of IGF-IR (Figure 7A). However, IGF-IR down-regulation eliminates exogenous increase in β1 protein levels suggesting that the stability of β1 is dependent on IGF-IR, reinforcing our previous observation that in the absence of IGF-IR, β1 is rapidly metabolized through proteasomal degradation. Both IGF-IR and β1 receptors seem to co-exist in a functional complex and stabilize each other's expression in cancer cells. As shown in Figure 7B, exogenous induction of β1 significantly promotes cancer cell proliferation. Concurrent IGF-IR down-regulation reduces β1 levels and thus significantly stalls cell proliferation suggesting that β1 functions are intrinsically dependent on IGF-IR expression in prostate cancer cells. Thus, it could be speculated that both IGF-IR and β1 receptors concertedly modulate JNK signaling in response to radiation.

## DISCUSSION

Using a novel hypofractionated radiation schedule that effectively blocks prostate tumor growth in TRAMP mice, we show that blocking JNK signaling using a JNK1, 2 and 3 inhibitor (SP600125), counteracts the effects of therapeutic radiation and leads to tumor growth and progression, in a β1 integrin-dependent manner.

Here we have tested a hypofractionated radiation scheme where a 50 Gy total dose of radiation was administered in 10 Gy fractions for 5 consecutive days, to approximate the hypofractionated approach in patients. This radiation schedule effectively suppresses tumor growth in β1^wt^ mice and also in β1^pc-/-^ mice which carry a conditional ablation of β1 integrins in the prostatic epithelium. However, inhibition of JNK phosphorylation negates the therapeutic effect of hypofractionated radiation in β1^pc-/-^ /TRAMP but not β1^wt^ /TRAMP mice. This is consistent with the notion that β1 integrin-dependent signaling determines the functional role of JNK as it relates to radiation response of the prostate epithelium.

Our study delineates a JNK-mediated signaling pathway, which modulates tumor growth upon irradiation in a differential manner dependent on β1 expression. It is widely accepted that ionizing radiation activates multiple signal transduction pathways, including the JNK/SAPK cascade, which transduce death signals from the cell membrane to the nucleus. Since β1 integrins suppress JNK activation induced by radiation [3], SP treatment, as expected, did not affect radiation-induced tumor suppression in β1^wt^ mice. Our results are in contrast with a previous report where inhibition of β1 integrins in head and neck carcinoma cells was reported to be associated with down-regulation of JNK signaling leading to radiosensitization [4]. However, there is substantial evidence demonstrating a pro-apoptotic role of JNK in response to radiation [26-29], which is consistent with our results. Although integrin interaction with the cytoskeleton is likely to mediate JNK activation, the association of β1 with the cytoskeletal protein, filamin A [30] is not likely to explain our results since filamin A binds stress signaling kinases MKK4 and MKK7 and is known to enhance JNK activation [31] whereas β1 integrins suppress radiation-dependent JNK signaling.

A unique feature of irradiated and SP-treated β1^pc-/-^ tumors is the up-regulation of FAK expression and auto-phosphorylation which is not observed in β1^pc-/-^ tissues from mice with functional JNK signaling or in β1^wt^ tissues where JNK is inhibited. FAK auto-phosphorylation at tyrosine 397 (FAK^pY397^) exposes a site for Src, which leads to Src-dependent phosphorylation of FAK at tyrosines 576 (FAK^pY576^) and 577 (FAK^pY577^) leading to maximal adhesion-induced FAK activation [32]. FAK expression has been reported to be enhanced in all stages of prostate tumorigenesis, to regulate anti-tumor immunity and integrin-dependent radioresistance [33-36]. Recently, nuclear FAK was reported to regulate immunomodulatory functions and inhibit anti-tumor immunity in cancerous squamous epithelial cells by regulating chemokine/cytokine and ligand receptor networks [36], which is consistent with our results. FAK activity has further been implicated in DNA damage induced NF-*k*B activation and production of cytokines from endothelial cells leading to chemoresistance [37]. Similarly, β1 integrin-dependent FAK signaling was reported to elicit faster cell attachment rates and reduced adhesion strength in taxol-resistant ovarian cancer cells [38]. The authors report that adhesion strength is dependent on FAK. Besides, faster attachment rates and reduced adhesion strength, in these cells, correlate with increased β1 integrin expression and decreased focal adhesion formation, respectively. In addition, drug-tolerant microenvironments are known to be correlated with high β1 integrin/FAK signaling in melanoma cells [39]. Our data indicating high FAK expression and phosphorylation associated with tumor progression upon radiation, are consistent with these studies and collectively highlight a central role of FAK in therapeutic resistance.

Activation of AKT has been reported earlier as an important predictor of the probability of PSA failure and a marker of clinically aggressive prostate cancer [40]. There is a significant interest in developing effective strategies to target this pathway [41]. In our studies, however, AKT activation in irradiated prostate tissues does not change upon JNK inhibition ruling out a significant functional contribution of JNK-dependent AKT activation in prostate cancer development in SP-treated β1^pc-/-^ TRAMP mice. Finally, although sustained FAK expression and activity have been reported to be essential for androgen-independent formation of neuroendocrine carcinoma [23], we observe significant chromogranin expression in TRAMP tissues that is not altered by either JNK inhibition or β1 abrogation.

Furthermore, our earlier report showed that abrogation of IGF-IR in prostate cancer cells enhances the sensitivity to radiation in a clonogenic assay [25]. Here we show that β1 integrins promote cell proliferation partly by enhancing the expression of IGF-IR underscoring the importance of β1/IGF-IR functional synergy. Using *in vitro* model systems of prostate cancer we have demonstrated a functional crosstalk between β1 integrins and IGF-IR and shown that these two vital receptors regulate each others' expression [25]. In the present report, we further demonstrate that exogenous β1 integrins up-regulate IGF-IR expression leading to enhanced cell proliferation. This is consistent with our previous findings where abrogation of IGF-IR led to reduced β1 levels *via* proteasomal degradation and enhanced radiation sensitivity of prostate cancer cells [3, 25]. The identification of aberrant signaling pathways broaden the current concept of radiation sensitivity in exploring multi-targeting molecular agents and aid in the development of novel therapeutic approaches.

In summary, we demonstrate that inhibition of JNK reactivates the growth of irradiated tumors, in a differential manner that depends on β1 integrins, and promotes FAK expression and activity. These findings have implications for the future design of combination therapies encompassing ionizing radiation and signal transduction modifiers.

## MATERIALS AND METHODS

### Reagents and antibodies

SP600125 (SP) was purchased from LC Laboratories. Murine monoclonal (m) antibodies (Abs) against the following antigens were used: human β1, TS2/16 (ATCC); β1, clone-18; JNK1/JNK2 (BD Pharmingen); c-Src (Cell Signaling). Rabbit polyclonal Abs against the following antigens were used: IGF-IR (IGF-IR-β sc713); AKT; FAK; ERK1/2 (Santa Cruz); chromogranin (Invitrogen); FAK^pY397^, Src^pY416^, JNK^pT183, pY185^, AKT^pS473^ and AKT (Cell Signaling). Non-immune rabbit IgG was purchased from Pierce. Alexa Fluor 488 goat anti-rabbit IgG was purchased from Invitrogen.

### Cell lines

LNCaP prostate cancer cells were purchased from ATCC and authenticated by the supplier. Cells were grown at 37°C and 5% CO2 in RPMI-1640 supplemented with 5% FBS and 1%, each of sodium pyruvate, HEPES and non-essential amino acids.

### Mice

TRAMP mice carrying conditional ablation of β1 (β1^pc-/-^ /TRAMP) and those expressing wild type β1 (β1^wt^ /TRAMP) were generated as described earlier [3]. Care of animals was in compliance with standards established by the office of laboratory animal welfare, Department of Health and Human Services at NIH. Experimental protocols were approved by the Institutional Animal Care and Use Committee, Thomas Jefferson University.

### SP injection and hypofractionated irradiation

SP was suspended in PP mixture comprising of 30% PEG-400, 20% polypropylene glycol, 15% cremophor, 5% ethanol and 30% saline [12]. At the age of 20 weeks, β1^pc-/-^ /TRAMP and β1^wt^ /TRAMP mice were subjected to IP injections of vehicle PP (150μl) or inhibitor SP (30mg/kg), administered 3 times / week for 10 weeks. Two hours after the first IP injection with either PP or SP, the lower pelvis of the mice was irradiated with 10 Gy radiation as a part of hypofractionated radiation regimen that consisted of 10 Gy fractions (total dose 50 Gy) administered for 5 consecutive days. Animals were anesthetized with Ketamine-Xylazine-Acepromazine mixture (0.125 ml/100g of body weight),(Ketamine, 80 mg/ml; Xylazine 5 mg/ml; Acepromazine, 1.6 mg/ml) providing 25-30 minutes sedation, prior to being placed in malleable lead shielding. The shield ensures that upper body including upper gastrointestinal tract is protected. Lower pelvises of mice were irradiated using a PanTak 310keV X-ray machine at 0.25mm Cu plus 1mm Al added filtration, at 125 cGy/min. Mice were euthanized at the age of 35 weeks for studying tumor progression. Prostate isolation was performed using a dissection stereomicroscope SZX10 (Olympus). Prostate tissues were weighed and fixed for histopathologic analysis. A small amount of tissue was frozen for immunoblotting analysis. Lungs, liver and lymph nodes were fixed for studying metastasis.

### Immunohistochemistry (IHC)

IHC analysis was carried out as reported earlier [3]. Lungs, liver and lymph nodes from mice were processed in the same manner and stained with H&E for studying metastasis. Histological analysis of prostate and metastases was performed by Dr. Peter McCue. Total FAK levels were analyzed in paraffin-embedded formalin-fixed tumor sections of β1^pc-/-^ /TRAMP mice by IHC as reported earlier for other molecules [42], with the exception of using biotin-streptavidin-amplified peroxidase immunodetection system with DAB kit (Invitrogen).

### Immunofluorescence and confocal microscopy

Immunofluorescence was carried out as described earlier [43]. Staining with an Ab to chromogranin was performed by incubation of tissue samples with primary Abs (1:100) for 1 hour at RT, followed by incubation with Alexa Fluor 488 goat rabbit IgG (1:250) for 20 minutes at RT.

### Transient transfection

Transfection of cells with siRNA oligonucleotides (Thermo Scientific) was performed as previously described [44]. To -regulate IGF-IR, the sequences of sense strands of duplex siRNAs used are as follows: IGF-IR-siRNA: 5′-CGACUAUCAGCAGCUGAAGUUdTdT-3′; control IGF-IR-siRNA: 5′-GAAGUCGACGACUAUCAGCU UdTdT-3′ [25].

### Cell proliferation assay

LNCaP cells were transfected with either empty vector pBJ1 or recombinant β1-pBJ1 plasmid together with either control siRNA or IGF-IR siRNA [25]. Cells were trypsinized 24 hours after transfection and plated in triplicate sets in fresh growth medium at 1.5×10^4^ cells/well in 6-well plates. Cells for growth assay were harvested at day 3 and day 6 after plating. Cells were trypsinized and pellets resuspended in 500μl of PBS followed by live-cell counting using hemocytometer. In parallel, cells were plated in 10 cm dishes to evaluate expression changes by immunoblotting.

### Immunoblotting (IB)

IB was performed on tumor and cell lysates as reported earlier [25, 43].

### Statistical analysis

Wilcoxon-Mann-Whitney test was used to compare the median tumor weight. Interquartile range and individual data points were plotted to compare the tumor mass between vehicle and SP-treated β1^pc-/-^ /TRAMP and β1^wt^ /TRAMP groups. Statistical analysis for proliferation assay was performed using two-tailed t-test.
